# Effect of Furnace Gas Composition on Characteristics of Supersonic Oxygen Jets in the Converter Steelmaking Process

**DOI:** 10.3390/ma13153353

**Published:** 2020-07-28

**Authors:** Liujie Yao, Rong Zhu, Yixing Tang, Guangsheng Wei, Kai Dong

**Affiliations:** 1 School of Metallurgical and Ecological Engineering, University of Science and Technology Beijing, Beijing 100083, China; 13521835960@163.com (L.Y.); tyx123615@163.com (Y.T.); weigsh_0418@126.com (G.W.); dongk123123@126.com (K.D.); 2 Beijing Key Laboratory of Research Center of Special Melting and Preparation of High-End Metal Materials, University of Science and Technology Beijing, Beijing 100083, China

**Keywords:** supersonic oxygen jet, numerical simulation, combustion experiment, furnace gas composition

## Abstract

During the converter steelmaking process, the presence of supersonic oxygen jets can provide oxygen to high-temperature metal baths that promotes chemical reactions in the bath, accelerates the smelting rhythm, and facilitates a uniform distribution of the ingredients in the bath. In this paper, a computational fluid dynamics (CFD) model with combustion reactions is established and compared to the results of combustion experiment. This paper studies the behavior and fluid flow characteristics of supersonic oxygen jets under different environmental compositions under a steelmaking temperature of 1873 K. This validated CFD model can be used to investigate the effect of furnace gas on supersonic oxygen jet characteristics during the converter steelmaking process. The results indicate that the composition of furnace gas has an impact on the characteristics of the oxygen jet. Specifically, as the carbon monoxide (CO) volume fraction increases, the high velocity region of supersonic oxygen jet increases, and the high temperature and the high turbulent kinetic energy regions expand.

## 1. Introduction

Top and bottom blowing converter steelmaking, which is the most important method of steelmaking, is employed worldwide. Top-blowing systems are usually realized by using a top-blowing oxygen lance. Oxygen passing through a Laval tube-shaped oxygen lance forms a supersonic jet, where the velocity of the supersonic jet is around Mach 2.0 [1]. When the high-velocity jet comes into contact with the melt in a molten pool, molten pool stirring is achieved, and the dynamic conditions of the molten pool are improved, and an oxidizing atmosphere is formed in the furnace [2]. The main components of converter furnace gas are carbon monoxide, carbon dioxide, and nitrogen [3]. Among them, carbon monoxide and carbon dioxide mainly emerge from decarburization, whereas the main source of nitrogen is bottom-blowing nitrogen. During the actual production process, as the smelting progresses, the composition of the converter gas continuously changes.

In view of the important role of supersonic oxygen jets, many scholars have conducted research on their characteristics. The main research methods are computation fluid dynamics (CFD) models, hydraulics experiments, and combustion tests [4,5,6,7]. Alam et al. [8] used a CFD model to investigate the effects of high-temperature environments on the characteristics of supersonic jets. They found that found that the potential core length of the jet increased significantly under high-temperature conditions. Lebon et al. [9] and Sumi et al. [10] obtained similar experimental results. Li et al. [11] used CFD models and water model experiments to study the energy transfer between the supersonic jet and the molten pool, it was found that about 50% of the total energy of the supersonic jets could be utilized to stir high-temperature melts. Yang et al. [12] used water model experiments to study the effect of the inclination of the center of the Laval tube to form the oxygen lance on the supersonic jet characteristics. The results showed that the staggered arrangement of the Laval tubes was conducive for the supersonic jet to stir the molten pool and to reduce the splash of the droplets. Zhao et al. [13] proposed a supersonic combustion coherent jet and used numerical simulation and combustion experiments to study the characteristics of supersonic jets. The results show that the supersonic combustion coherent jet could be stably burned under high temperature environments, and the combustion flame is beneficial in protecting the flow characteristics of the main oxygen jet because the supersonic length of the coherent jet is increased. Liu et al. [14] used numerical simulation and combustion experiments to study the characteristics of the lance structure on supersonic jets. The results show that the coherent lance is beneficial in increasing the surface area of the impact pit during the steelmaking process because the supersonic jet has better stirring ability for the molten pool and improves the efficiency of the reaction. Li et al. [15] used CFD models to study the coalescence characteristics of supersonic jets formed by lances for converter steelmaking. The results show that the ambient temperature, the number of nozzles and the nozzle angle have an effect on the convergence of multiple supersonic jets, and as the ambient temperature increases, the number of nozzles decrease, and the jet velocity and dynamic pressure decay appear to be delayed.

In summary, many researchers use numerical simulation, water model and combustion experimental methods to study the characteristics of supersonic jets, such as the pressure and temperature of the environment and the structure of the lance. However, there have been few reports on the characteristics of the furnace gas composition on the supersonic jet, which will lead to deviations in the laboratory research results when applied to the actual production process and will affect the quality of the crude steel produced by the converter. Therefore, this paper studies the characteristics of supersonic jets with different furnace gas components.

In this research, we first determined that the main gas components in the furnace gas were: carbon monoxide (CO), carbon dioxide (CO_2_), oxygen (O_2_) and nitrogen (N_2_). Then, according to the change law of the furnace gas during the converter steelmaking process, five kinds of furnace gas states were proposed. A series of numerical simulations and combustion experiments were studied in regards to the characteristics of supersonic jets. During the combustion experiment, the axial velocity and the axial total pressure of the jet were measured to verify the accuracy of the numerical simulation. Then, the velocity distribution, total temperature distribution and turbulent kinetic energy distribution of the supersonic jet formed by the Laval tube and the oxygen lance were analyzed according to the numerical simulation results.

## 2. Experimental

### 2.1. Experimental Equipment

**Figure 1:**
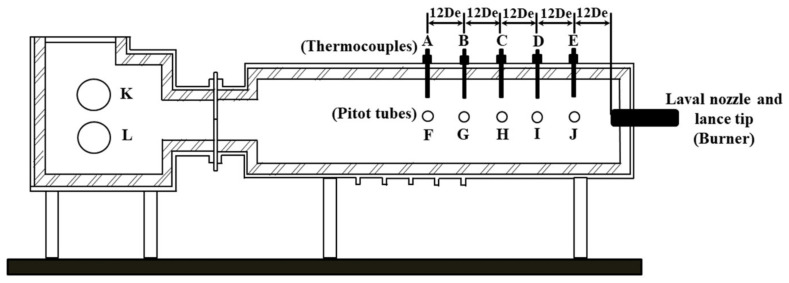
Schematic view of the experimental apparatus.

**Figure 2:**
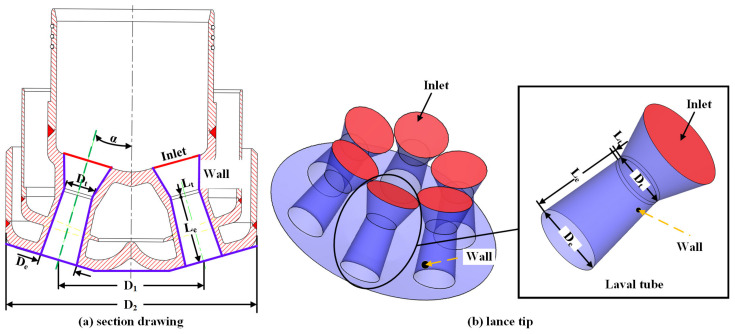
Structure of the (**a**) cross-sectional drawing of the lance tip. (**b**) Lance tip.

A high temperature furnace was used to conduct the combustion experiment in order to explore the effect of furnace gas composition on the characteristics of supersonic oxygen jets in this article. Zhao et al. [13] used a similar device to study the stirring ability of the supersonic combustion coherent jet in an electric furnace molten pool. Liu et al. [14] used a cylindrical combustion device to study the influence of the lance structure on electric furnace steelmaking. During the high temperature experiment, they used pitot tubes and thermocouples to measure pressure and temperature, respectively [16,17,18,19,20]. Therefore, this article used Pitot tubes and thermocouples to measure pressure and temperature. The structures of the high temperature furnace and the lance tip are presented in Figure 1 and Figure 2.

In the experiment preparation stage, thermocouples, which had a top that was 1 cm from the wall of the furnace, were installed at points A, B, C, D, and E, and the distance between the two monitoring points was 12 De, where De is the diameter of the exit of the Laval nozzle. Since, it was difficult to maintain uniform and stable temperatures in the high temperature furnace during the experiment, the average value of the temperature of thermocouples at each point was taken as the monitoring value of the temperature. Pitot tubes, which were applied to measure pressure, were installed at points F, G, H, I, and J. During the combustion experiment, the total pressure after the shock (*P*_0*a*_) and the static pressure before the shock (*P_b_*) could be measured, and Equations (2) and (3) could be used to solve the total pressure before the shock (P_0b_). Points K and L were inlets for the ambient gas. A burner heated with methane was installed on the right side of the device to increase the temperature of the combustion furnace, as presented in Figure 1. At the beginning of the experiment, the combustion furnace was heated to the required temperature with the burner, and at the same time, the ambient gas was added to the furnace from points K and L. After the desired temperature was reached, the burner was removed, and a lance with a water cooling system was installed in its place. Then, the lance was supplied with oxygen and the experimental data was measured [16,17,18,19,20]. In this article, the temperatures at points A, B, C, D, and E were 1873 ± 16 K, 1873 ± 14 K, 1873 ± 10 K, 1873 ± 24 K, and 1873 ± 19 K, respectively.

Figure 2a shows the cross-section view of the lance passing through two Laval tubes. Figure 2b is a three-dimensional perspective view of the established lance model that includes six uniformly distributed Laval tubes. As presented in Figure 2, the red and purple parts are the inlet and wall of the lance, respectively. The diameter of the throat and exit are D_t_ and D_e_, respectively, whereas the lengths of the extension and throat are L_e_ and L_t_, respectively. D_1_ denotes the distance between the central axes of the Laval tubes; D_2_ is the outer diameter of the lance; and α is the angle between the nozzle holes. The specific dimensions of the lance tip apparatus are presented in Table 1.

### 2.2. Measurement Method

During the experiment, the temperature of the ambient air was 1873 K. Schemes 1 and 2, which are the volume fraction of carbon monoxide (CO), were 0% and 20% were conducted for safety, because carbon monoxide is a flammable gas with an explosion limit of 12.5–74.2% [21]. A Pitot tube was installed at the monitoring point during the experiment, which was used to monitor the total pressure after the shock (*P*_0*a*_) and the static pressure before the shock (*P_b_*).

The isentropic law of a compressible gas was applied to calculate the velocity of the oxygen jet as:

(1) $$\nu =\sqrt{\frac{2\kappa }{\kappa -1}RT_{0}\left[1-{\left(\frac{p_{b}}{p_{0a}}\right)}^{\frac{\kappa -1}{\kappa }}\right]}$$

(2) $$\frac{P_{0a}}{P_{b}}=\frac{{\left(\frac{\kappa +1}{2}Ma^{2}\right)}^{\frac{\kappa }{\kappa -1}}}{{\left(\frac{2\kappa }{\kappa +1}Ma^{2}-\frac{\kappa -1}{\kappa +1}\right)}^{\frac{1}{\kappa -1}}}$$

(3) $$\frac{P_{0b}}{P_{b}}={\left(1+\frac{\kappa -1}{2}Ma^{2}\right)}^{\frac{\kappa }{\kappa -1}}$$

where *v* denotes the velocity of the measuring point, m/s; *p*_0*a*_ and *T*_0_, the total pressure after shock and total temperature of the ambient at the measuring point, Pa and K, respectively; *P_b_* and *P*_0*b*_ are the static pressure and the total pressure before the shock at the monitoring point Pa, respectively; *R* is the ideal gas constant N·m·kg^−1^·K^−1^; and *κ* is the heat capacity ratio.

## 3. Numerical Modeling

### 3.1. Assumptions

The following assumptions were made in our numerical simulations:

(1) Oxygen is an ideal gas;
(2) The supersonic jet is a three-dimensional, steady-state, and nonisothermal Newtonian fluid;
(3) The nozzle wall is smooth. A nonslip boundary condition was used to the walls, and a standard wall function was applied to calculate the mean velocity near the wall.

### 3.2. Governing Equations

#### 3.2.1. Equation of Continuity

The equation of continuity is given as:

(4) $$\frac{\partial \rho }{\partial t}+\nabla \cdot \left(\rho \vec{\nu }\right)=S_{m}$$

where *ρ* denotes the density of the gas, kg/m^3^, *ρ* = *p*/RT, and $\vec{\nu }$ denotes the velocity of the fluid, m/s. The source (*S_m_*) is the mass added to the continuous phase from the dispersed second phase, which is zero in this study.

#### 3.2.2. Momentum Conservation Equation

The conservation of momentum equation is:

(5) $$\frac{\partial }{\partial t}\left(\rho \vec{\nu }\right)+\nabla \cdot \left(\rho \vec{\nu }\vec{\nu }\right)=-\nabla p+\nabla \cdot \left\{\mu \left[\left(\nabla \vec{\nu }+\nabla {\vec{\nu }}^{T}\right)-\frac{2}{3}\nabla \cdot \vec{\nu }I\right]\right\}+\rho \vec{g}+\vec{F}$$

where *p* is the static pressure, Pa; *μ* is the molecular viscosity, Pa∙s; $\nabla {\vec{\nu }}^{T}$ describes the effect of volume dilation; I is a unit tensor; $\rho \vec{g}$ is the gravitational body force, N; and $\vec{F}$ is the external body force, N.

#### 3.2.3. Turbulence Model

In this study, the standard k-ε turbulence model was used [22,23]. The expressions of the turbulent kinetic energy (*k*) and dissipation rate (*ε*) are obtained from the following equations:

(6) $$\frac{\partial }{\partial t}\left(\rho k\right)+\frac{\partial }{\partial x_{i}}\left(\rho k\nu_{i}\right)=\frac{\partial }{\partial x_{j}}\left[\left(\mu +\frac{\mu_{t}}{\sigma_{k}}\right)\frac{\partial k}{\partial x_{j}}\right]+G_{K}+G_{b}-\rho \varepsilon -Y_{M}+S_{k}$$

(7) $$\frac{\partial }{\partial t}\left(\rho \varepsilon \right)+\frac{\partial }{\partial x_{i}}\left(\rho \varepsilon \nu_{i}\right)=\frac{\partial }{\partial x_{j}}\left[\left(\mu +\frac{\mu_{t}}{\sigma_{\varepsilon }}\right)\frac{\partial \varepsilon }{\partial x_{j}}\right]+C_{1\varepsilon }\frac{\varepsilon }{k}\left(G_{k}+C_{3\varepsilon }G_{b}\right)-C_{2\varepsilon }\rho \frac{\varepsilon^{2}}{k}+S_{\varepsilon }$$

where *ν_i_* is the velocity of the fluid in the direction I, m/s; *G_k_* and *G_b_* represent the generation of turbulent kinetic energy due to the mean velocity gradients and buoyancy, respectively, *J*; *Y_M_* is the contribution of the fluctuation dilation in the compressible turbulence to the overall dissipation rate; *S_k_* and *S_ε_* are user-defined source terms (which are zero in this article); *C_1ε_*, *C_2ε_*, and *C_3ε_* are 1.44, 1.92, and 0.8, respectively; *σ_k_* and *σ_ε_* are 1.0 and 1.3, respectively [24]; and *μ_t_* is the turbulent viscosity, which is obtained by combining *k* and *ε* as follows:

(8) $$\mu_{t}=\rho C_{\mu }\frac{k^{2}}{\varepsilon }$$

where *C_μ_* is a constant with a value of 0.09.

#### 3.2.4. Energy Equation

The energy equation is given as:

(9) $$\frac{\partial }{\partial t}\left(\rho E\right)+\nabla \cdot \left[\vec{\nu }\left(\rho E+p\right)\right]=\nabla \cdot \left[k_{eff}\nabla T-\sum_{j}h_{j}\vec{J_{j}}+\left({\overset{=}{\tau }}_{eff}\cdot \vec{\nu }\right)\right]+S_{h}$$

where *k_eff_* is the effective conductivity, $k_{eff}=k+\frac{c_{p}\mu_{t}}{{Pr}_{t}}$; $\vec{J_{j}}$ is the diffusion flux of species *j*, $k_{eff}\nabla T$, $-\sum_{j}h_{j}\vec{J_{j}}$, and $\left({\overset{=}{\tau }}_{eff}\cdot \vec{\nu }\right)$ represents the energy transfer due to conduction, species diffusion, and viscous dissipation, respectively; *S_h_* is the heat produced by chemical reactions and any other volumetric heat sources; and E is defined as:

(10) $$E=\sum_{j}\left[Y_{j}\cdot \left(\int_{T_{ref}}^{T}c_{p,j}dT\right)\right]-\frac{p}{\rho }+\frac{\nu^{2}}{2}$$

where *Y_j_* is the mass fraction of *j*, *C_p,j_* is the temperature-dependent constant pressure specific heat capacity of species *j*, and *T_ref_* is 298.15 K, as used for the pressure-based solver.

#### 3.2.5. Species-Conservation Equation

In this paper, a species model was used to solve the conservation equations for all chemical species. The local mass fraction of each species, *Y_j_*, was predicted through the solution to the convection–diffusion equation for the *j*th species. The conservation equations are presented below:

(11) $$\frac{\partial }{\partial t}\left(\rho Y_{j}\right)+\nabla \cdot \left(\rho \vec{\nu }Y_{j}\right)=-\nabla \cdot \vec{J_{j}}+R_{j}$$

(12) $$\vec{J_{j}}=-\left(\rho D_{j,m}+\frac{\mu_{t}}{Sc_{t}}\right)\nabla Y_{j}-D_{T,j}\frac{\nabla T}{T}$$

where *R*_j_ is the net rate of the j-species produced by chemical reactions, $\vec{J_{j}}$ represents the diffusion flux of species *j*, *D_j,m_* represents the mass diffusion coefficient for the *j*_th_ species in the mixture, *D_T,j_* is the thermal diffusion coefficient, and *Sc_t_* is the turbulent Schmidt number, which was 0.7 in this paper.

#### 3.2.6. Combustion Model

The eddy dissipation (ED) model was employed to study the effects of the composition of the ambient gas on the characteristics of the supersonic oxygen jet [25,26]. The model assumes that the reaction rate is controlled by the turbulence, and the time dependence of the chemical reactions was ignored. The ED model was chosen because it is computationally cheap. Within the ED model, the net rate of the production of species *j* is due to reaction *r*; *R_j,r_* means the molar rate of species *j* in reaction *r* is given by the smaller of the two equations as follows:

(13) $$R_{j,r}=\nu_{j,r}^{\prime }M_{\omega ,j}A\rho \frac{\varepsilon }{k}\min_{R}\left(\frac{Y_{R}}{\nu_{j,r}^{\prime }M_{\omega ,R}}\right)$$

(14) $$R_{j,r}=\nu_{j,r}^{\prime }M_{\omega ,j}AB\frac{\varepsilon }{k}\left(\frac{\sum_{p}Y_{P}}{\sum_{i}^{N}\nu_{i,r}^{\prime \prime }M_{\omega ,i}}\right)$$

where $\nu_{j,r}^{\prime }$ is the stoichiometric coefficient for reactant *j* in reaction *r*; $\nu_{i,r}^{\prime \prime }$ is the stoichiometric coefficient for product *j* in reaction *r*; $M_{\omega ,j}$ is the molecular weight of species *j*; *A* and *B* are empirical constants equal to 4.0 and 0.5, respectively; *k* is the kinetic energy of the turbulence; *ε* is the dissipation rate; and *Y_P_* and *Y_R_* are the mass fractions of any product species *P* and a particular reactant *R*, respectively.

#### 3.2.7. Discrete Ordinates Radiation Model

As the temperature of the ambient medium was 1873 K, the polyatomic gas (CO_2_) and the asymmetric diatomic gas (CO) in the ambient gas have certain radiating and absorbing capabilities, and so CO_2_ and CO must be considered when analyzing and calculating the total radiative heat transfer. The weighted sum of the gray gas model (WSGGM) is often used to define the temperature of a medium and the emissivity related to the concentration of the medium [27]. In this work, the discrete ordinates radiation model (DO model) was used [28,29,30,31] and applied with the WSGGM model to obtain the results of the combustion experiment [29]. The radiative transfer equation for absorbing, emitting, and scattering mediums at position $\vec{r}$ in direction $\vec{s}$ is presented below:

(15) $$\nabla \cdot \left[I\left(\vec{r},\vec{s}\right)\vec{s}\right]+\left(a+\sigma_{s}\right)I\left(\vec{r},\vec{s}\right)=an^{2}\frac{\sigma T^{4}}{\pi }+\frac{\sigma_{s}}{4\pi }\int_{0}^{4\pi }I\left(\vec{r},{\vec{s}}^{\prime }\right)\Phi \left(\vec{s},{\vec{s}}^{\prime }\right)d\Omega^{\prime }$$

where $\vec{r}$ and $\vec{s}$ are the position and direction vectors, respectively; ${\vec{s}}^{\prime }$ and *s* are the scattering direction vector and path length, respectively; *a* is the absorption coefficient, L·mol^−1^·cm^−1^; *n* is the refractive index; *σ_s_* is the scattering coefficient; *σ* is the Stefan–Boltzmann constant (5.669 × 10^−8^ W/m^2^/K^4^); *I* is the radiation intensity, W/sr; *T* is the local temperature, K; Φ is the phase fraction; and Ω′ is the solid angle, sr.

### 3.3. Solution Method

**Figure 3:**
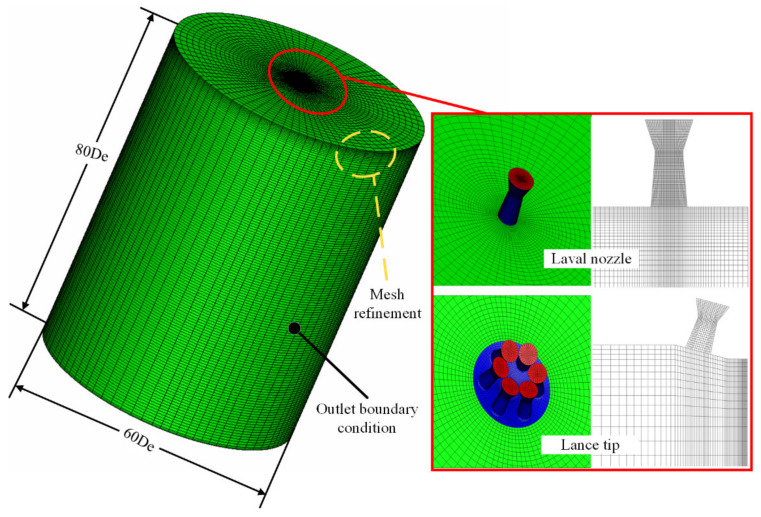
Detailed grid arrangement of the computational fluid dynamics (CFD) model.

In this study, the CFD simulations were performed using ANSYS Fluent 17.0 [15,16]. During the numerical simulations, a three-dimensional geometric model was used. Figure 3 shows the detailed grid arrangement of the CFD model, and the green part is the outlet boundary condition.

In the spatial discretization, a hexahedral structured mesh was applied, where the mesh near the exit of the Laval tube was optimized to improve the accuracy of the simulation. For the Laval tube, the circumferential was divided into 64 equal parts, and the extension section and the throat section were divided into 65 and four equal parts, respectively, and there were 110,959 nodes inside the Laval tube. For the Laval tube of the lance tip, the circumferential was divided into 24 equal parts, and the extension section and throat section were divided into 17 and two equal parts, respectively, and there were 45,570 nodes among the six Laval tubes of the lance tip. The calculation domain was a cylindrical area with a length of 80 De and a radius of 30 De, where, as described previously, De is the exit diameter of the Laval tube, as presented in Figure 3. The lance tip inlet was the mass flow inlet, and the wall boundary condition at this position was adopted. The other boundary of the calculation domain was defined at the pressure outlet. The specifics of the boundary conditions used in the simulations are presented in Table 2. The compositions of the ambient gas studied here are presented in Table 3.

In this paper, the numerical simulations were based on a pressure-based solver [15], where the pressure-velocity coupling equation was solved using the coupling algorithm (COUPLED algorithm method). The least-squares cell-base was used to compute the gradient, and the standard discrete form of the pressure was adopted. The density, momentum, turbulent kinetic energy, turbulent dissipation rate, and energy were all solved using the second-order upwind scheme. When the residual error of the energy was less than 10^−6^, the residual errors of other variables were less than 10^−5^ [32], and when the difference between the inlet and outlet mass flows was less than 10^−3^, the calculation was considered to have reached convergence.

### 3.4. Grid Independency Test

**Figure 4:**
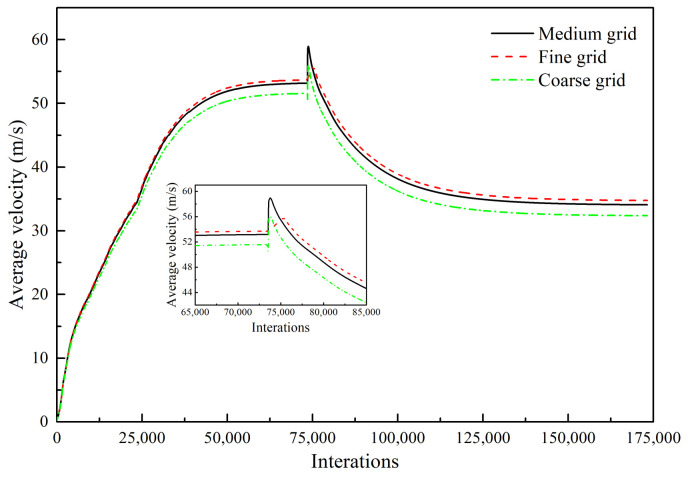
Average velocity of the longitudinal section as a function of three grid resolutions (Scheme 2).

In the numerical simulations, to explore the degree of dependence of the calculated results on the grid resolution, three grid resolutions were applied to monitor the velocity of the center plane of a supersonic jet formed by one Laval tube under the condition of the composition of the ambient gas in Scheme 2: 697,800 grid points (fine-resolution grid), 550,784 grid points (medium-resolution grid), and 330,730 grid points (coarse-resolution grid) [12,13,14,15,16,17,18]. Figure 4 indicates the variation of the area-weighted average velocity of the longitudinal section for the three grids. For the three grid resolutions, the average velocity of the longitudinal section with the interations changes according to the same pattern. As the interations increases, the area-weighted average velocity gradually increases, and the average velocity reaches a stable value when the interations reach 50,000. The combustion model is turned on when the interations reach approximately 74,000. Therefore, the average velocity shows a peak, and the velocity gradually decreases and finally remains stable. The average velocity error of the coarse grid relative to the medium grid is 5% larger, whereas the average velocity error value is 2% larger for the medium grid relative to the fine grid. As the number of grid points increases, the calculation time doubles. Therefore, under the condition of ensuring accurate calculations, balanced by calculation efficiency, we used a medium-resolution grid for the numerical simulations.

## 4. Results and Discussion

### 4.1. Combustion Experiment and CFD Model Validation

**Figure 5:**
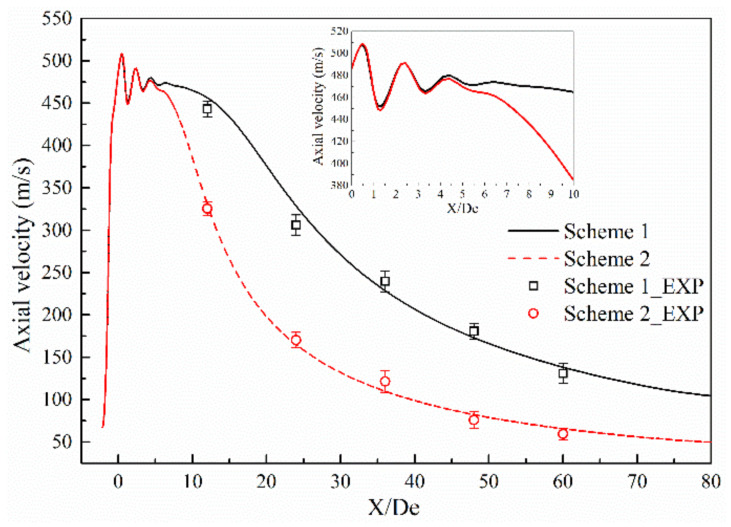
Comparison of axial velocity between the combustion experiment results and the numerical simulation results.

**Figure 6:**
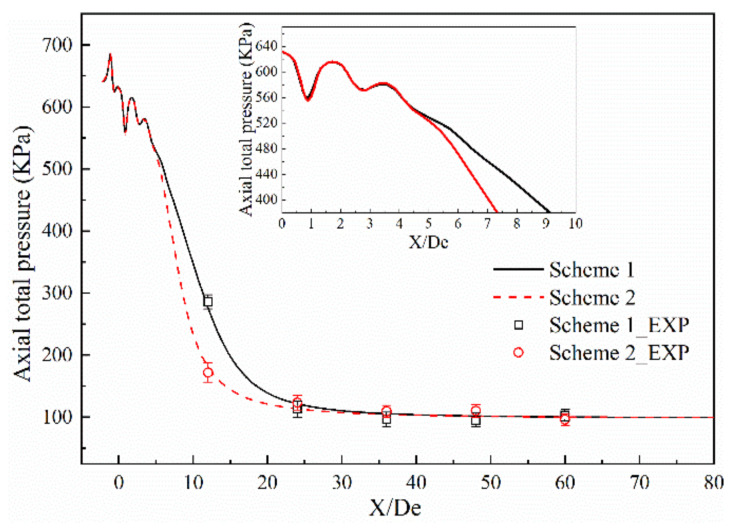
Comparison of axial total pressure between the combustion experiment results and the numerical simulation results.

Figure 5 and Figure 6 show the axial velocity and axial total pressure distribution of the supersonic oxygen jet in the numerical simulation and combustion experiment of Schemes 1 and 2 [13,14,15,16,17,18,19,20]. Table 4 and Table 5 shows the average and standard deviation of the axial velocity and the axial total pressure at the monitoring point, respectively. For all schemes, the axial velocity and axial total pressure repeatedly fluctuates at the exit of the Laval tube. In the design process of the Laval tube, the outlet pressure of the Laval tube is 101,325 Pa and the temperature of the oxygen is 298 K. However, as oxygen is preheated and a combustion reaction occurs, the outlet pressure and temperature of the nozzle changes. Therefore, shocks are formed as the supersonic oxygen jet incorrectly expands. After the supersonic oxygen jet repeatedly fluctuated, the axial velocity of the jet stabilized, and the axial total pressure rapidly decreased to become equal to the pressure of the ambient air.

A comparison of the combustion experiment results and numerical simulation results is presented in Figure 5 and Figure 6, where the black solid line and the red dashed line indicate the numerical simulation results of Schemes 1 and 2, respectively, and □ and ○ represent the experimental results under the same schemes. The maximum error values of axial velocity of the combustion experiments of Schemes 1 and 2 are 12.20% and 12.70%, respectively. The maximum absolute errors of axial velocity between the results of numerical simulation and combustion experiment of Schemes 1 and 2 are 6.38% and 9.65%, respectively. The maximum error values of the total axial pressure of the combustion experiments of Schemes 1 and 2 are 15.30% and 14.50%, respectively. The maximum absolute errors of axial total pressure between the results of the numerical simulation and combustion experiments of Schemes 1 and 2 are 9.36% and 9.11%, respectively.

This comparison shows that the results of the numerical simulation are in good agreement with the results of combustion experiment, and the rationality of the model selection for the numerical simulations is verified.

While good agreement was observed between the simulations and experiments, there were some small differences that were probably caused by the following.

(1) In the numerical simulations, oxygen was considered to be an ideal compressible gas and the ambient gas was an ideal gas. However, during the combustion experiments, the properties of both the oxygen and ambient gas exhibited a tendency to deviate from those of an ideal gas [33].
(2) During the numerical simulations, the temperature in the combustion furnace was uniform, but in the combustion experiment, the temperature in the furnace was not always uniformly distributed.
(3) During the numerical simulations, the ED model was used for the combustion process, which ignored the chemical kinetic rate, and it was considered that the combustion reaction occurred immediately after mixing.
(4) In the numerical simulations, heat exchange between the lance tip and the cooling water was not considered.

### 4.2. Velocity Distribution

**Figure 7:**
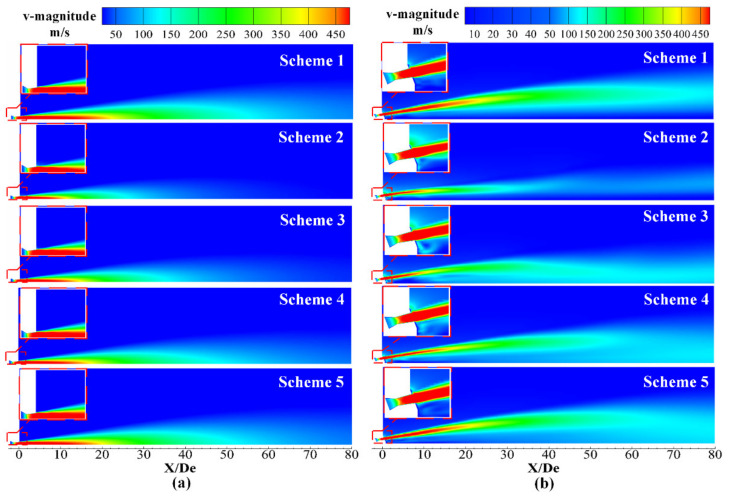
Velocity distribution of the supersonic jet along the longitudinal section. (**a**) Laval nozzle and (**b**) lance tip.

Figure 7 presents the velocity distribution of the supersonic jet along a longitudinal section of the Laval nozzle and lance tip, where red indicates high-velocity regions and blue indicates low-velocity regions. Inspection of Figure 7a revealed that the jet was in a high-velocity region near the outlet of the Laval nozzle, and as the jet moved away from the outlet, the velocity of the jet gradually decreased. At the same time, it was found that the high-velocity regions of Schemes 2–5 gradually increased, but they were all smaller than those observed in Scheme 1. This was due to the combustion reaction of the supersonic oxygen jet with the combustible gas in the furnace gas, which affected the velocity distribution of the supersonic jet of the Laval tube.

**Figure 8:**
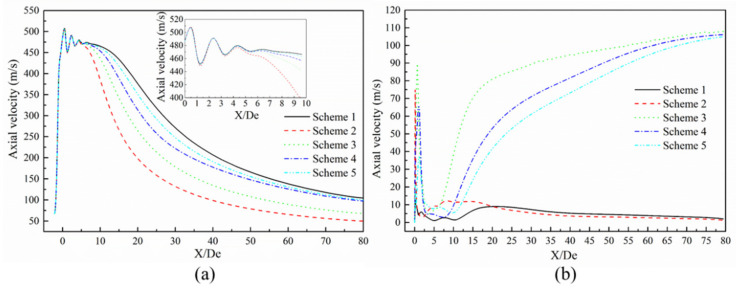
Axial velocity distribution of the supersonic jet. (**a**) Laval nozzle and (**b**) lance tip.

It can be seen from Figure 7b that the velocity distributions of Schemes 2–5 and Scheme 1 show significant differences, which was due to the combustion reaction between the supersonic oxygen jet, the ambient gas and the mutual interaction between the various streams. By comparing Schemes 2–5, it can be seen that, with the change in the ambient gas composition of the furnace gas, the velocity distribution of the supersonic jet had a significant difference in the radial direction, which was mainly due to the blending between the supersonic jet and the ambient gas. The details will be explained in Section 4.4. The convergence between the streams can be judged by observing the velocity distribution near the axis [15]. The velocity distributions around the axes of Schemes 1 and 2 are smaller than those of Schemes 3–5, so the degree of convergence between the jets of Schemes 1 and 2 is small. It can be seen that the jets in Scheme 3 converge near 15 De, and the Schemes 4 and 5 converge near 25 De and 30 De, respectively. The specific axis velocity distribution can be seen in Figure 8b.

**Figure 9:**
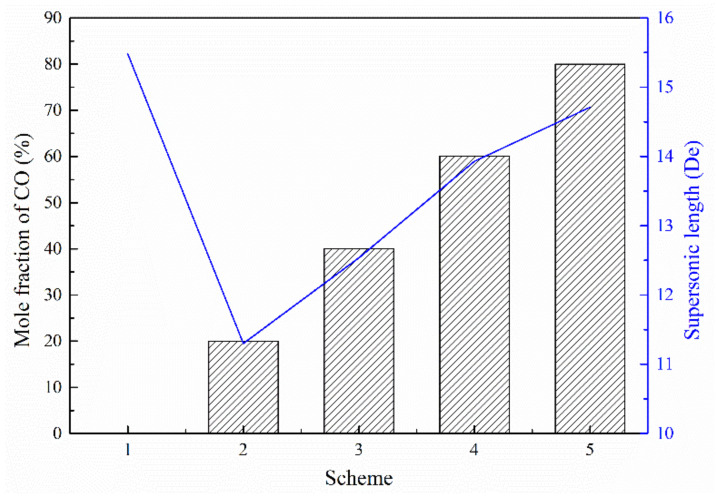
Supersonic length of the Laval nozzle.

Figure 8 presents the axial velocity distribution of the supersonic jets produced by the Laval nozzle and the lance tip. As presented in Figure 8a, the axial velocity of the jet at the outlet of the Laval nozzle oscillates and then gradually decreases as the jet propagates away. The decay of the axial velocity in the range of 10 to 40 De was greater than the decay of the axial velocity in the range of 40 to 80 De. According to the statistical analysis of the supersonic length of the Laval nozzle, the supersonic length of Scheme 1 was found to be 15.48 De. The supersonic lengths of Schemes 2–5 were 0.73, 0.81, 0.90, and 0.95 times the length found in Scheme 1, respectively—as presented in Figure 9. Comparing Scheme 1 and other schemes, it can be seen that the combustion reaction between the ambient gas and the oxygen jet has an effect on the velocity distribution of the supersonic jet, and for Schemes 2–5, as the mole fraction of the ambient gas composition changes, the length of the supersonic jet gradually increases. This phenomenon confirms that the composition of the ambient gas has an influence on the velocity distribution of the supersonic jet. The reason should be that the supersonic oxygen jet is related to the combustion of carbon monoxide in the ambient gas.

As shown in Figure 8b, the axial velocity distribution of Scheme 2 is similar to that observed in Scheme 1, where the latter scheme does not consider combustion. The distributions for Schemes 3, 4, and 5 exhibit similar behavior. For Schemes 1 and 2, the axial velocity reaches a maximum near 20 and 8 De, respectively, and the axial speed decreases slightly afterwards. For Schemes 3–5, the axial speed first increases to a large extent and reaches a maximum near 19.64, 29.92, and 32.01 De, respectively. Then, the slope of the axial speed gradually decreases with the change in the axial position. It can be seen that the supersonic axial velocity distribution at the outlet of the lance tip varies as a function of the furnace gas molar fraction. This is mainly because the supersonic oxygen jet has a combustion reaction with CO in the furnace gas, which affects the convergence of the jet under different conditions of the furnace gas composition [15].

**Figure 10:**
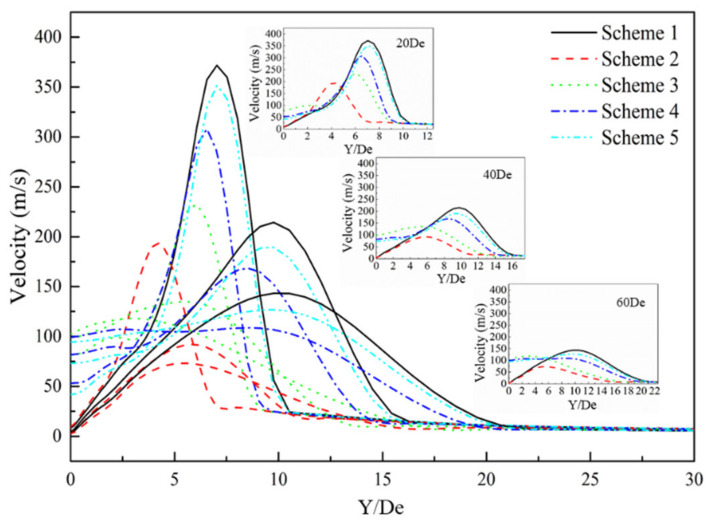
Radial velocity distribution of the supersonic jet of the lance tip.

Figure 10 presents the radial velocity distribution of the supersonic jet produced by the lance tip at different positions. It can be seen that the radial velocity of the jet exhibits a trend of increasing first and then decreasing to zero. At positions close to the Y/De = 0, the speeds in Schemes 1 and 2 show a consistent pattern, whereas those of Schemes 3–5 exhibit similar behavior. The radial velocity of Scheme 2 first reaches a maximum, followed by maxima for Schemes 3–5. Schemes 1 and 5 are almost the same, which indicates that when the carbon monoxide in the furnace gas and oxygen jets undergo a combustion reaction, the velocity distribution of the supersonic jet in the radial direction will be affected, and the magnitude of the influence is inversely proportional to the size of the carbon monoxide mole fraction in the environment.

### 4.3. Total Temperature Distribution

**Figure 11:**
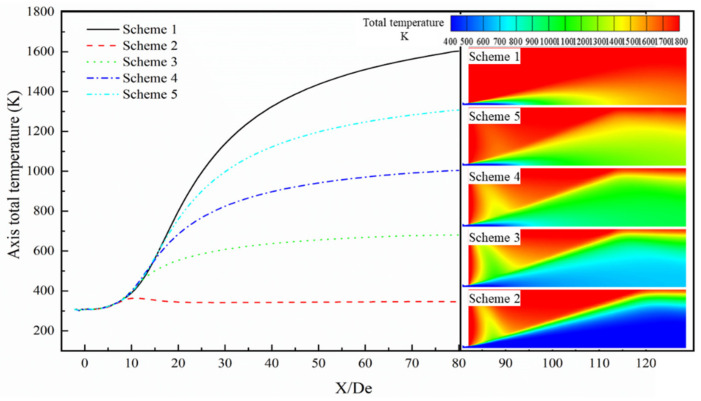
Axis total temperature distribution of the supersonic jet produced by the Laval nozzle.

**Figure 12:**
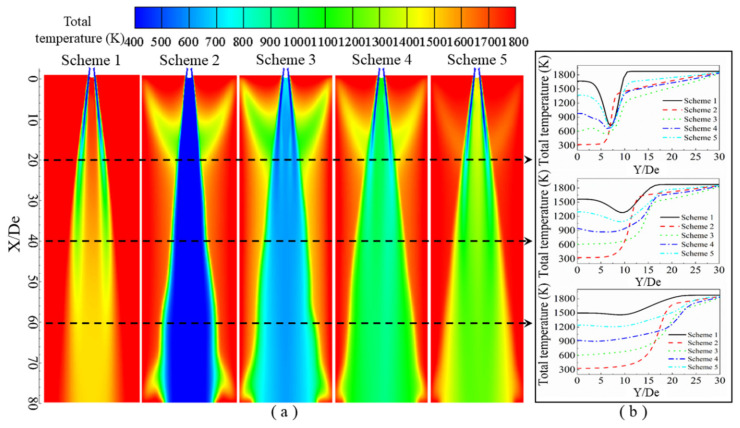
Total temperature distribution of the supersonic jet produced by the lance tip. (**a**) Total temperature contours along the longitudinal section, and (**b**) total temperature distribution as a function of the radial position.

Figure 11 and Figure 12 present the total temperature distribution curves of the axial and the longitudinal sections of the supersonic jet produced by the Laval tube and the lance tip, respectively. In the figures, red indicates high-temperature regions and blue indicates low-temperature regions.

The total temperature is the sum of the static and dynamic temperature of the airflow, which reflects the conversion of energy between thermal and kinetic energies [34]. The total temperature of the supersonic gas jet remains stable near the outlet of the Laval tube and then exhibits an increasing trend. Finally, due to energy conversion between the jet and the ambient gas, the total temperature of the gas jet gradually stabilizes.

As presented in Figure 11, the total temperature of the jet as a function of the axial position, for the five schemes when the axial distance is less than 3.8 De, shows that the total temperature of the jet hardly changes with position, and the temperature of the jet is equal in the different schemes. In the axial position range of 3.8 to 8.9 De, the total temperatures of the different schemes are almost equal, and the total temperature of the jet appears to gradually increase with the axial position. When the axial position is greater than 8.9 De, the total temperature as a function of the axial position increases gradually and then gradually tends to a constant temperature. This again proves that when the supersonic jet is ejected from the Laval tube, the oxygen jet and the furnace gas undergo a combustion reaction, and the combustion reaction affects the total temperature distribution of the supersonic jet. Further, the magnitude of the effect of the combustion reaction on the total temperature distribution is inversely proportional to the carbon monoxide mole fraction in the furnace gas. This phenomenon can be clearly observed from the cloud diagram of the total temperature distribution.

It can be seen from the contours of the total temperature distribution in Figure 12 that low-temperature zones are mainly located along the axis of the single jet, and the total temperature at the position between the gas streams is lower than the ambient temperature. Recall that Scheme 1 does not consider combustion reactions, and in this scheme, it can be observed that as the jet advances, the oxygen stream and ambient gas become mixed, so that the single jet gradually converges toward the jet’s axis, and the temperature of the oxygen in the jet gradually increases [16]. In comparing Schemes 2–5 with Scheme 1, it can clearly be observed that when the oxygen jet comes into contact with the ambient gas, a burning phenomenon occurs, so that a clear triangular area appears near 10 De. In Schemes 2–5, it can be observed that as the CO content in the ambient gas increases, the total temperature near the axis gradually increases, among which Scheme 2 has the lowest temperature, which is almost equal to the inlet oxygen temperature, and Scheme 5 has the highest temperature.

It can be seen from the distribution of the total temperature of the jet in the radial direction in Figure 12b that when the abscissa Y/De is 0, it corresponds to the axis position in the longitudinal section. At different radial positions, the total temperature of each scheme is at first a stable value, which then changes. When Y/De = 20, the total temperature of Scheme 1 rapidly decreases after a stable value and then rapidly increases until it reaches the ambient temperature. The total temperature of Scheme 2 rapidly increases after a stable value until it reaches that of the ambient temperature. For Schemes 3–5, the distance where the radial total temperature is at a stable value is very short. Then, the temperature gradually decreases and then rapidly increases to ambient temperature. When Y/De = 40, the radial total temperature distribution of each test scheme is similar to that of Y/De = 20, but the ranges over which changes occur are smaller. When Y/De = 60, the total radial temperature of each scheme does not decrease with a change in the radial position, but instead they gradually increase to the ambient temperature. The reason for this is that near Y/De = 0, the total temperature is at a stable value mainly because a negative-pressure zone forms between the streams of the oxygen jet, so its internal temperature can maintain a relatively stable value. As the radial distance increases, the total temperature rapidly decreases to a minimum value, that is, at the center of the single jet, which then rapidly increases, because the oxygen jet mixes with the ambient gas, causing the jet temperature to increase.

It can be seen from the total temperature distribution of the supersonic jet that combustion occurs when the supersonic jet contacts the furnace gas, and the mole fraction of carbon monoxide in the furnace gas affects the degree of combustion. The flame formed by the combustion can effectively prevent the mixing of the supersonic oxygen jet and furnace gas, so a low total temperature zone appears between the streams.

### 4.4. Turbulent Kinetic Energy Distribution

**Figure 13:**
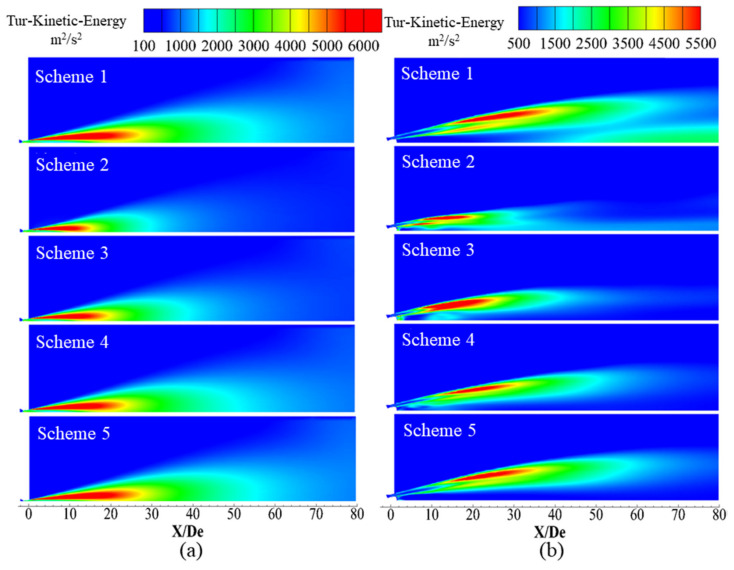
Turbulent kinetic energy distribution of the supersonic jet along the longitudinal section. (**a**) Laval nozzle and (**b**) lance tip.

Figure 13 presents the turbulent kinetic energy distribution of supersonic jets formed by the Laval tube and the oxygen lance, where red indicates regions of high turbulent kinetic energy and blue indicates regions of low turbulent kinetic energy.

Turbulent kinetic energy is an important parameter that characterizes the average kinetic energy per unit mass associated with a vortex in a turbulent flow. At the same time, turbulent kinetic energy is a measure of the degree of mixing between fluids, where it is generally believed that the greater the turbulent kinetic energy, the greater the degree of mixing. In this work, turbulent kinetic energy is utilized to reflect the mixing characteristics of the supersonic oxygen jets with the ambient gas.

Figure 13 presents the turbulent kinetic energy distribution of a longitudinal section of the supersonic oxygen jet in different environmental gas compositions under a high-temperature environment. For high-temperature and CO-containing environments, the oxygen jet reacts with the ambient gas and becomes enveloped by a combustion flame. It can be seen from Figure 13 that for Schemes 2–5, as the volume fraction of CO in the ambient gas increases, the turbulent kinetic energy of the jet boundary layer gradually increases, but they are all less than the turbulent kinetic energy observed in Scheme 1. This phenomenon has an impact on the density of the ambient gas and the oxygen jet density ratio, thereby affecting the size and distribution of the turbulent kinetic energy.

## 5. Conclusions

In previous studies, the influence of furnace gas composition on supersonic oxygen jet characteristics has rarely been reported. In this paper, the volume fraction of carbon monoxide, carbon dioxide, oxygen and nitrogen was adjusted to simulate the furnace gas. Based on numerical simulations and combustion experiments that measured the effects of different furnace gas components on the characteristics of supersonic oxygen jets, the results are conducive to further deepening people’s understanding of the characteristics of supersonic jets, conducive to the understanding of supersonic jet by steelmakers and to converter production, and having a certain impact on the design of oxygen lances. The following conclusions can be obtained:

(1) An oxygen supersonic jet CFD model has been developed that takes into account the flow characteristics and combustion effects of compressible gas in detail. In the numerical simulations, the ED model was used to perform the calculations. The maximum absolute errors of axial velocity and total pressure between the results of numerical simulation and combustion experiment are 9.65% and 9.36%, respectively. It was found that the numerical simulation results were in good agreement with the combustion test data.
(2) In this paper, it is found that carbon monoxide in the furnace gas will react with the oxygen jet. Therefore, it will affect the coalescence, velocity distribution, total temperature distribution and turbulent kinetic energy distribution of the supersonic jet, and the degree of influence is inversely proportional to the carbon monoxide mole fraction. Specifically, as the volume fraction of carbon monoxide in the ambient atmosphere increased, the area of the low total temperature regions gradually decreased, the turbulent kinetic energy in the boundary layer gradually increased, and the high velocity region of the supersonic oxygen jet extended, the length of the supersonic jet is extended from 11.53 De to 15.01 De with the volume fraction of carbon monoxide increasing from 20% to 80%.
(3) In the traditional design process of the supersonic oxygen lance, the influence of the combustible gas in the furnace gas on the oxygen jet is not taken into account. Therefore, in the actual production processes, the actual results often deviate from the laboratory results. Based on the results of this work, we believe that the design process of the oxygen lance should be revised, depending on the composition of the furnace gas. This is an important direction for subsequent research.

## Figures and Tables

**Table 1:** Geometry of the lance tip.

Parameter	Values
D_t_	54.1 mm
D_e_	70.3 mm
L_t_	5 mm
L_e_	92.5 mm
D_1_	235 mm
D_2_	406 mm
α	16°

**Table 2:** Specifications of the boundary conditions.

Name of Boundary	Type of Boundary Conditions	Values
Inlet	Mass flow rate	3.61 kg/s
	Initial gauge pressure	767,606 Pa
	Total temperature	308 K
Wall	Standard wall function	298 K
	No-slip	
Outlet	Gauge pressure	98,100 Pa
	Backflow total temperature	1873 K

**Table 3:** The compositions of the ambient gas.

Item	Mole Fraction (%)				M_mixture_ (g/mol)	ρ (kg/m^3^)
Scheme	CO	CO_2_	O_2_	N_2_		
1 ^a^	0	0	20	80	28.8	1.1640
2	20	20	0	60	31.2	1.2600
3	40	15	0	45	30.4	1.2275
4	60	10	0	30	29.6	1.1950
5	80	5	0	15	28.8	1.1625

^a^ The ambient gas of Scheme 1 was regarded as air during the combustion experiments.

**Table 4:** Velocity and total pressure at the monitoring points.

Mearing Point	F		G		H		I		J	
	v m/s	P KPa	v m/s	P KPa	v m/s	P KPa	v m/s	P KPa	v m/s	P KPa
Scheme 1	456.78	277.59	327.05	121.27	230.46	106.05	172.70	101.92	137.29	100.39
Scheme 2	332.05	181.31	163.87	113.21	110.82	104.60	82.12	101.57	65.16	100.30
Scheme 1-EXP	443.07	285.91	306.19	112.71	239.68	96.12	180.80	95.16	130.91	103.61
Scheme 2-EXP	325.40	171.97	170.42	123.52	121.51	109.36	75.92	110.51	59.51	96.27

**Table 5:** Standard deviation of velocity and total pressure at the monitoring points of the combustion experiment.

Mearing Point	F		G		H		I		J	
	v	P	v	P	v	P	v	P	v	P
Scheme 1-EXP	11.24	11.23	11.90	12.56	12.20	11.71	9.15	11.07	11.63	9.07
Scheme 2-EXP	8.27	15.66	9.15	12.07	12.70	9.64	10.02	10.37	6.60	9.66
